# Salivary miRNAs and cytokines associated with diagnosis and prognosis of oral squamous cell carcinoma

**DOI:** 10.3389/fcell.2025.1531016

**Published:** 2025-01-22

**Authors:** Yuxiao Qin, Xiaodan Dong, Bo Li

**Affiliations:** Department of Oral Anatomy and Physiology, Jilin Provincial Key Laboratory of Oral Biomedical Engineering, Hospital of Stomatology, Jilin University, Changchun, China

**Keywords:** oral squamous cell carcinoma, diagnosis and prognosis, salivary biomarker, miRNAs, cytokines

## Abstract

Oral squamous cell carcinoma (OSCC) is the most common malignant tumour in the oral and maxillofacial region. Early diagnosis can significantly improve the 5-year survival rate of patients with OSCC. Therefore, it is extremely important to differentiate OSCC patients early, easily and quickly. Human saliva contains a variety of components that can be used as biomarkers for the diagnosis and prognosis of OSCC. Studies have shown that salivary microRNAs (miRNAs) and cytokines are closely associated with the progression of OSCC. The aim of this review is to summarize the research progress of salivary biomarkers (miRNAs and cytokines) in the past 3 years, and to explore the possibility of using miRNAs and cytokines to improve the diagnosis and prognosis of OSCC.

## 1 Introduction

Oral squamous cell carcinoma (OSCC) is the most common oral cancer and one of the deadliest malignancies with a 5-year survival rate of about 50% (Bray et al., 2024). Patients with OSCC are usually asymptomatic in the early stages and have insufficient understanding of risk factors including smoking, HPV infection, and alcohol (Ahmadi et al., 2019), which means they are typically diagnosed at an advanced stage, leading to poor prognosis and increased mortality. It is well known that HPV-positive (HPV+) OSCCs have a better prognosis than HPV-negative (HPV-) ones (Kumar et al., 2024). The prognostic difference between HPV+ and HPV- OSCCs is due to the impact of HPV infection on both microRNAs (miRNAs) and inflammatory cytokines. HPV + OSCCs exhibit distinctive miRNA profiles, which influence tumour progression and immune responses (Christianto et al., 2022). Additionally, HPV infection modulates the levels of inflammatory cytokines, possibly contributing to the unique tumour microenvironment observed in HPV + OSCCs (Castellano et al., 2022). Early diagnosis of OSCC is very important to improve its prognosis (Jeng et al., 2001; Guha et al., 2014; Cristaldi et al., 2019; Worthington et al., 2023).

The definitive approach for diagnosing OSCC involves clinical oral examination coupled with histological assessment of tissue samples from the affected area (Lopez-Escamez and Perez-Carpena, 2024). Currently, the emphasis in cancer diagnosis lies on identifying minimally invasive and cost-effective methods (Wang et al., 2017). Liquid biopsy (LB) represents a minimally invasive technique to obtain biological and dynamic information about cancer from a patient’s body fluid samples (Poulet et al., 2019). It facilitates the real-time monitoring of cancer progression. LB-related studies have been extended to a variety of physiological fluids, including urine and saliva, as well as to a variety of molecular entities such as DNA, RNA and extracellular vesicles (Alix-Panabières et al., 2023). Saliva biopsies have been proposed as an alternative method for cancer detection and prognosis due to their ease of collection (Patel et al., 2022).

Saliva contains cytokines, RNA and DNA molecules, tissue derivatives, and other components that contribute to disease diagnosis. The collection of saliva for identifying diagnostic markers represents a direct, non-invasive, convenient, and economical method. This potential has been thoroughly investigated over the past few decades. Recent studies have explored the feasibility of salivary biomarkers in diagnosing the onset and progression of OSCC (Kawahara et al., 2016; Chen et al., 2017; Sivadasan et al., 2020). Notably, salivary factors exhibit stable and disease-specific expression patterns in human peripheral blood and bodily fluids (Kunicki et al., 2024; Sharma et al., 2024; Tan et al., 2024), while miRNAs can serve as ideal biomarkers for this purpose (Chen et al., 2008; Ryu et al., 2023; Fattahi et al., 2024; Ko et al., 2024; Shaterabadi et al., 2024).

This review aims to emphasize advancements in salivary miRNAs and cytokines research over the last 3 years. The investigation of the possible effectiveness of salivary biomarkers (cytokines and miRNAs) in enhancing the diagnosis and prognosis of OSCC has been conducted over the past 3 years.

## 2 Salivary miRNAs associated with OSCC diagnosis and prognosis

miRNAs consist of 20–24 nucleotides and were originally discovered in the roundworm *Caenorhabditis elegans* (Lee et al., 1993). miRNAs have the capacity to regulate gene expression post-transcriptionally. miRNAs can be isolated from cells, tissues and body fluids such as serum, plasma, tears or urine (Weber et al., 2010), and can also be selectively packaged in extracellular vesicles (Betel et al., 2008) (Table 1).

**TABLE 1:** Salivary miRNAs associated with OSCC diagnosis and prognosis.

Biomarker	Discoveries	Clinical relevance	Reference
miRNA-200 miRNA-34 miRNA-24 miRNA-124 miRNA-21 miRNA-136 miRNA-3928 miRNA-31 miRNA-486-5p miRNA-10b-5p miRNA-106a	Associated with OSCC stage and histological classification and/or grading	Biomarkers for early diagnosis and monitoring Novel therapeutic targets to help develop intervention strategies against OSCC	Faur et al. (2022), Tarrad et al. (2023), Vageli et al. (2023), Bahrami et al. (2024), Farshbaf et al. (2024), Shalaby et al. (2024), Wu et al. (2024)
miRNA-21 miRNA-181b miRNA-184 miRNA-106a miRNA-146a miRNA-155 miRNA-3928	Malignant diseases such as OPMD and precancerous lesions associated with OSCC	Biomarkers for the diagnosis of OSCC and other related malignant diseases Simultaneous detection of these miRNAs can examine the risk of OSCC	Di Stasio et al. (2022), Garg et al. (2023), Mehdipour et al. (2023), Farshbaf et al. (2024)
miRNA-3928 miRNA-186 miRNA-506-3p miRNA-153-3p miRNA-203a-5p miRNA-34a miRNA-643 miRNA-876-5p miRNA-149-3p miRNA-18a miRNA-340-5p miRNA-145-5p	Role of tumour-inhibiting	These miRNAs can inhibit OSCC progression and may have therapeutic potential for OSCC	Deng et al. (2022), Ding et al. (2022), Fu et al. (2022), Kim et al. (2022), Long et al. (2022), Ou et al. (2022), Shen et al. (2022), Cui et al. (2023), Zhang et al. (2023), Zhao et al. (2023), Zhou and Jin (2023), Farshbaf et al. (2024)
miRNA-19a miRNA-1307-5p miRNA-21-5p miRNA-21-3p miRNA-205-5p	Oncogenes facilitating the initiation and advancement of OSCC	Provides a potential method for identifying OSCC and associated malignant lesions	Jadhav et al. (2022), Patel et al. (2022), Chen et al. (2023), Huang et al. (2023), Vageli et al. (2023)

### 2.1 miRNAs: tumour biomarkers

In 2008, Lawrie et al. compared the levels of tumour-associated miRNAs in the sera of diffuse large B-cell lymphoma (DLBCL) patients with those of healthy controls, and found that the levels of miRNAs in the patients were significantly higher than those in the controls, which suggested for the first time that circulating miRNAs are expected to be used as non-invasive markers for DLBCL (Lawrie et al., 2008). Subsequently, by studying miRNA stability, site differences, etc., scholars have shown that miRNAs of tumour origin detected in plasma or serum have great potential as circulating biomarkers for detecting common human cancer types (Mitchell et al., 2008). Then, miRNAs have been suggested as diagnostic and prognostic biomarkers for the treatment of several tumours, including breast and ovarian cancers, and OSCC (Zahran et al., 2015; Tung et al., 2020; Sempere et al., 2021; Coskunpinar et al., 2023). In recent years, the analysis of abnormal miRNAs profile levels in OSCC patients has been reviewed (Ghafouri-Fard et al., 2020; Manzano-Moreno et al., 2021; Li et al., 2024; Romani et al., 2024; Sanesi et al., 2024). Moreover, in this year’s Nobel Prize in Physiology or Medicine, the discovery of miRNAs was honored, which may represent another new high point in the upcoming research related to miRNAs. In summary, by analyzing miRNAs information in cancer patients, new biomarkers can be developed for OSCC clinical diagnosis. In addition, they are significant regulators of gene transcription and may be potential biomarkers for forecasting clinical outcomes of OSCC.

miRNAs can either increase or inhibit the expression of target genes by directly binding to the mRNAs of those genes. They also influence the stability of mRNA (Hudder and Novak, 2008). Aberrant miRNA regulation can significantly contribute to cancer development (Shi et al., 2008). The expression of some miRNAs is associated with the dysregulation of oncosuppressors or oncogenes and contributes to tumour development or suppression. miRNAs with oncogenic functions are upregulated and silence tumour-inhibiting genes that can facilitate cancer cell progression. Conversely, miRNAs with tumour-inhibiting functions are downregulated, thereby reducing the regulation of oncogenes and maintaining malignancy (Lee and Dutta, 2006). It is necessary to further elucidate the carcinogenic and tumour-inhibiting functions of different miRNAs and their specific mechanisms using new technologies such as bioinformatics or single-cell sequencing analysis, and establish the stable correlation between miRNAs and OSCC progression, which will help to identify specific biomarkers for the diagnosis and prognosis of OSCC.

### 2.2 Diagnostic value of salivary miRNAs for OSCC

The diagnostic value of salivary miRNAs in identifying diagnostic markers has been extensively studied in recent years, which will possess the important clinical significance and bring greater translational benefits with the deepening of research.

#### 2.2.1 Salivary miRNAs from OSCC patients

Recent studies have shown that salivary miRNAs can be used for early diagnosis and clinical staging of OSCC. Researchers and technicians are committed to improving the salivary miRNA analysis platform in sample collection and pretreatment, RNA extraction technology, detection methods, data analysis algorithms, and quality control systems (Yoshizawa and Wong, 2013; Fortuna et al., 2020; McKenna and Dubey, 2022). Vageli et al. reported statistically significant differences in salivary miRNA-21, miRNA-136 and miRNA-3928 expression between early tumours and healthy controls (*p* < 0.05) (Vageli et al., 2023) (Table 1). Bahrami et al. found that in saliva samples from OSCC patients, the expression levels of two biomarkers, miRNA-200 and miRNA-34, were reduced relative to healthy individuals, whereas the expression level of miRNA-24 was increased (Bahrami et al., 2024) (Table 1). Shalaby et al. showed that miRNA-124 expression was significantly decreased in the saliva of OSCC patients (*p* < 0.001) (Shalaby et al., 2024) (Table 1). Tarrad et al. claim that miRNA-106a expression helps to differentiate between OSCC grade II and grade III (Tarrad et al., 2023) (Table 1). Wu et al. developed a dual 3D nanorobot capable of rapidly, sensitively and specifically analyzing miRNA-31, a diagnostic biomarker for OSCC, in saliva samples (Wu et al., 2024) (Table 1). Scheurer et al. compared saliva from healthy and OSCC samples using different isolation techniques and identified 11 differentially expressed miRNAs (*p* < 0.05): miRNA-1183, 128-1-5p, 3646, 3648, 383-5p, 4300, 4638-5p, 486-5p, 5189-5p, 6076 and 6784-5p (Scheurer et al., 2024). Meanwhile, in the study by Momen-Heravi et al., it was found that of the more than 700 miRNAs that were analyzed, 13 were identified as being significantly dysregulated in saliva samples from patients with OSCC when compared to healthy controls. 11 of these were found to be significantly underexpressed (miRNA-136, -147, −1250, −148a, −632, −646, −668, −877), Conversely, two miRNAs (miRNA-24, -27b) demonstrated significant overexpression (*p* < 0.05) (Momen-Heravi et al., 2014). The diagnostic value of these miRNAs in the saliva of OSCC patients needs to be further verified, providing potential research directions for future studies.

#### 2.2.2 Salivary miRNAs from OSCC precancerous lesions

Furthermore, miRNA detection in saliva also exhibits the important diagnostic value for oral potentially malignant disease (OPMD) and precancerous lesions that may develop into OSCC. Garg et al. reported that miRNA-21 and miRNA-184 are biomarkers of OSCC and OPMD, where miRNA-21 was significantly increased in OSCC and OPMD, while miRNA-184 was significantly decreased (Garg et al., 2023) (Table 1). Di Stasio et al. noted that miRNA-181b showed upregulation (*p* = 0.006) in the saliva of patients in the OPMD high-grade dysplasia group, which was positively correlated with the degree of dysplasia and negatively correlated with OSCC (Di Stasio et al., 2022) (Table 1).

#### 2.2.3 Salivary miRNAs for OSCC differential diagnosis

In addition, some salivary miRNAs can be used for the differential diagnosis between OSCC and other oral diseases. Tarrad et al. suggested that low salivary miRNA-106a levels may indicate malignancy and may also be used to diagnostically differentiate OSCC from oral lichen planus (OLP) (Tarrad et al., 2023). Mehdipour et al. reported increased expression of miRNA-146a in saliva of OLP patients (*p* = 0.004) (Mehdipour et al., 2023) (Table 1). In addition, miRNA-155 was significantly upregulated only in saliva specimens from OLP patients (*p* = 0.009). Farshbaf et al. found that the expression level of miRNA-3928 in saliva of OLP patients (*p* = 0.01) was different from that of OSCC patients (*p* < 0.0001) compared to healthy individuals, and the expression level of miRNA-3928 in saliva of OSCC patients was markedly downregulated (Farshbaf et al., 2024) (Table 1). The above studies have shown that salivary miRNA-106a, 146a, 155, and 3928 may serve as potential biomarkers for the differential diagnosis between OSCC and OLP.

#### 2.2.4 Salivary exosomal miRNAs from OSCC patients

Exosomes are small, single-membrane, secreted organelles of ∼30 to∼200 nm in diameter that have the same topology as the cell and are enriched in selected proteins, lipids, nucleic acids, and glycoconjugates (Pegtel and Gould, 2019). Exosomes, which are one of the smallest extracellular vesicles released from cells, have been shown to carry different nucleic acids, including miRNAs (Krylova and Feng, 2023). The significant advantage of salivary exosomal miRNAs lies in convenient sampling and better stability. Salivary exosomal miRNAs can be extracted from a very small amount of saliva and have been successfully used to detect OSCC. Faur et al. noted that salivary exosomal miRNA-486-5p was raised, while miRNA-10b-5p was diminished in OSCC relative to healthy controls. miRNA-486-5p expression levels are elevated in stage II OSCC (Faur et al., 2022) (Table 1). Patel et al. found that the expression level of miRNA-1307-5p was significantly elevated in tissue and salivary exosome samples from individuals diagnosed with OSCC when compared to samples from individuals without cancer. From a clinical perspective, the upregulation of miRNA-1307-5p has been linked to a number of unfavourable outcomes, including patient survival, disease progression, aggressiveness, and chemotherapy resistance (Patel et al., 2022).

#### 2.2.5 Combined analysis of salivary miRNAs

It has also been shown that the combined analysis of multiple miRNAs in saliva is necessary to distinguish OSCC patients from healthy individuals. Scholtz et al. showed that a panel consisting of miRNA-345, miRNA-31-5p and miRNA-424-3p could be used to diagnose OSCC. In addition, IL-6 and miRNA-31 are synergistic in cancer stem cell activity, with higher specificity of salivary IL-6 for OSCC, and improved specificity by the combination of IL-6 mRNA and miRNA-31 as biomarkers (Scholtz et al., 2022). Meanwhile, Di Stasio et al. showed that salivary miRNA-27b and miRNA-181b can be used as potential biomarkers of oral dysplasia, and the combination of the two has implications for the diagnosis of the disease (Di Stasio et al., 2022) (Table 1). A more accurate diagnosis of OSCC would be achieved by using multiple miRNAs as a panel, with simultaneous corresponding changes, that is to say, panel changes.

### 2.3 miRNAs: tumour-inhibiting effect

**FIGURE 1:**
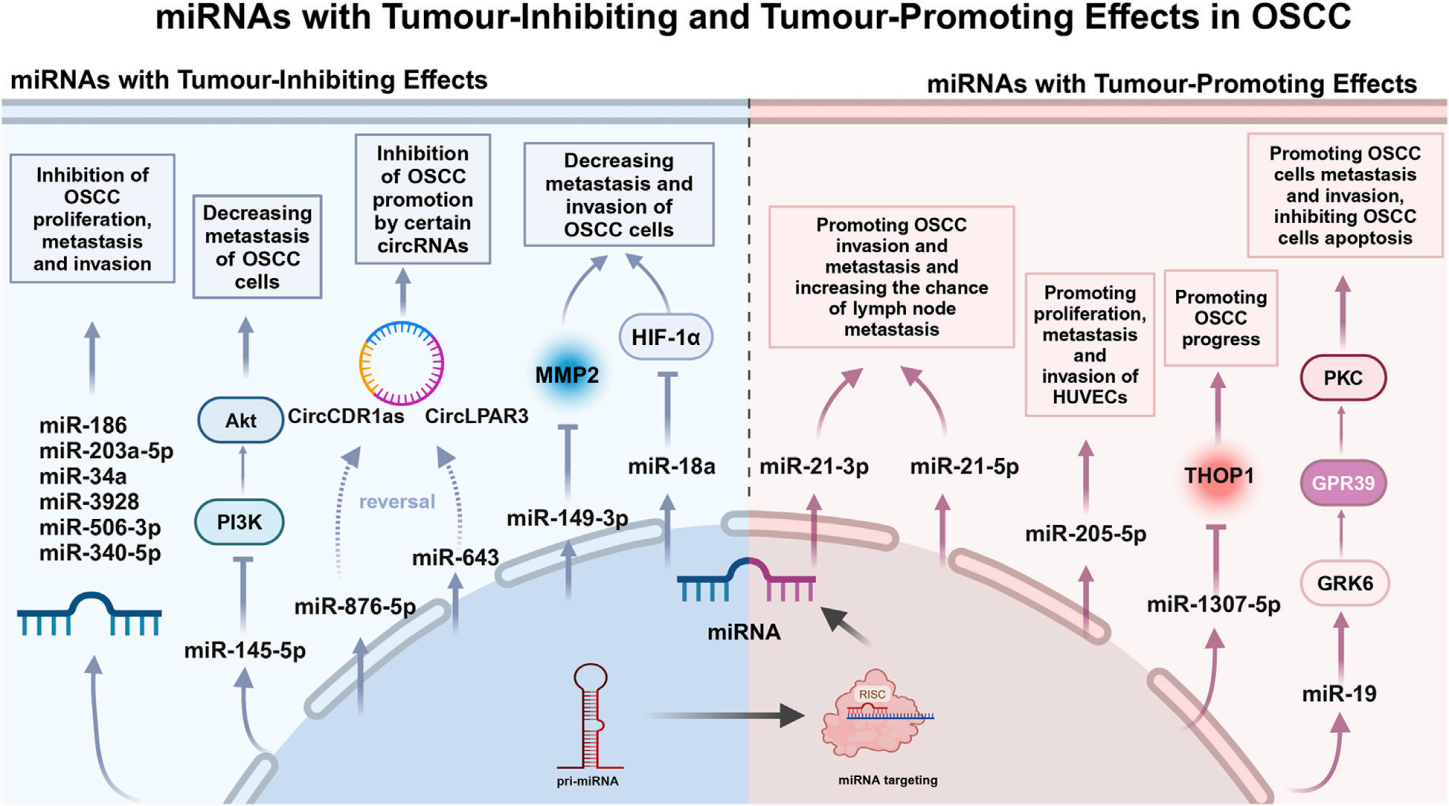
miRNAs with Tumour-Inhibiting and Tumour-Promoting Effects in OSCC.

In the past 3 years, a few studies have been conducted on the tumour suppressive effects of salivary miRNAs. Farshbaf et al. noted that miRNA-3928, which acts as a tumour-inhibiting in OSCC pathobiology, was significantly reduced in patient saliva (Farshbaf et al., 2024) (Table 1). Given the prospective tumour-inhibiting function of miRNA-3928 in OSCC, this miRNA may play an important role in early diagnosis, screening and targeted therapy of OSCC (Figure 1).

The extensive studies on tumour-inhibiting miRNAs in serum or tissue have been conducted by multiple research groups, which may provide the potential targets for future studies on tumour-inhibiting effects of salivary miRNAs. Zhao et al. declared that miRNA-186 reduces OSCC cell proliferation, migration and invasion and promotes apoptosis (Zhao et al., 2023) (Table 1). Ding et al. suggested that miRNA-506-3p retards OSCC cell progression at elevated concentrations (Ding et al., 2022) (Table 1). Long et al. identified miRNA-153-3p as a tumour-inhibiting in OSCC (Long et al., 2022) (Table 1). Zhang et al. reported that miRNA-203a-5p was elevated to inhibit OSCC cell invasion and migration (Zhang et al., 2023) (Table 1). Deng et al. identified miRNA-34a as an inhibitor of OSCC cell proliferation, migration and invasion (Deng et al., 2022) (Table 1). Numerous tumour-inhibiting miRNAs work by counteracting the expression of circular RNAs (CircRNAs) that facilitate cell proliferation. For example, miRNA-643 reverses the promotion of OSCC progression by CircLPAR3 (Fu et al., 2022) (Table 1). miRNA-876-5p mitigates the impact of increased circCDR1as on autophagy, cell cycle progression, proliferation, motility, and death in OSCC cells through its overexpression (Cui et al., 2023) (Table 1). Several miRNAs can exert tumour-inhibiting effects by regulating tumour-associated factors. Shen et al. reported inhibition of matrix metalloproteinase-2 (MMP-2) by miRNA-149-3p, which was associated with a reduction in the invasive process in OSCC cells (Shen et al., 2022) (Table 1). Kim et al. constructed a miRNA-18a mimic and observed that it inhibited HIF-1α expression. In addition, they found that it reduced the metastatic and invasive potential of OSCC cells (Kim et al., 2022) (Table 1). Ou et al. found that downregulation of miRNA-340-5p enhances invasion and proliferation of oral cancer cells (Ou et al., 2022) (Table 1). Zhou et al. showed that miRNA-145-5p inhibited the PI3K/AKT pathway and hindered cell migration (Zhou and Jin, 2023) (Table 1).

### 2.4 miRNAs: tumour-promoting effect

The mechanism of salivary miRNAs promoting OSCC progression has gradually become a research focus. Chen et al. reported that overexpression of miRNA-19a is regulated through the miRNA-19a/GRK6/GPR39/PKC signaling pathway and thereby inhibiting apoptosis and promoting cell migration and invasion. It is a key regulator of OSCC progression and provides a possible avenue for developing novel therapies targeting downstream signaling pathways (Chen et al., 2023) (Table 1). Patel et al. found that miRNA-1307-5p expression was elevated in tissues and salivary exosomes of patients with oral cancer, which may promote oral cancer progression through mechanisms such as inhibition of Thimet Oligopeptidase 1 (THOP1). Increased expression of this miRNA is associated with decreased patient survival, disease progression, invasiveness, and chemotherapy resistance, and may serve as a reliable prognostic marker for predicting adverse outcomes in oral cancer (Patel et al., 2022) (Table 1). Jadhav et al. found that miRNA-21 is an important oncogenic miRNA associated with tumour invasion and metastasis. Sensitivity and specificity analyses showed that the specificity of salivary miRNA-21-5p was 90.30%, while that of salivary miRNA-21-3p was 83.90% (Jadhav et al., 2022) (Table 1). miRNA-21 was significantly overexpressed in OSCC smokers compared to non-smokers (*p* = 0.006), suggesting an association between smoking and the oncogenic effects of OSCC (Vageli et al., 2023) (Table 1). Huang et al. suggested a significant increase of exosomal miRNA-205-5p in saliva of OSCC patients. It was shown to promote proliferation, metastasis and invasion of HUVECs (Huang et al., 2023) (Table 1). Therefore, the study on the pro-carcinogenic mechanism of miRNAs may provide the experimental basis and theoretical basis for the clinical diagnosis and prognosis of OSCC.

## 3 Salivary cytokines associated with OSCC diagnosis and prognosis

Salivary cytokines are important regulators involved in the inflammatory response of the body and the regulation of the tumour microenvironment. In OSCC patients, variations in salivary cytokine concentrations are tightly associated with tumour occurrence and their biological behavior. Studies have shown that dysregulation of specific cytokine levels is associated with the emergence of different cancer types and is considered an important biomarker for the diagnosis of tumours (Chechlinska et al., 2008; Kartikasari et al., 2021; Uchendu et al., 2023). Increasing research indicates that analyzing salivary cytokine levels can enhance the early identification of OSCC and assist doctors in formulating personalized treatment strategies (Ferrari et al., 2021). These findings offer novel insights into salivary cytokines for the diagnostic and prognostic evaluation of OSCC and may aid in the identification of new biomarkers.

### 3.1 TNF-α

TNF-α is recognized as a key factor in many inflammatory, viral, metabolic, and neoplastic diseases (van Loo and Bertrand, 2023). Meanwhile, the role of TNF-α can be seen in many human malignancies, including breast (Cruceriu et al., 2020), gastric (Zhao et al., 2010), pancreatic (Egberts et al., 2008), ovaries (Gupta et al., 2016), endometrial (Morgado et al., 2016), prostate (Tse et al., 2012), bladder (Sethi et al., 2012; Ting et al., 2021), colorectal (Li et al., 2016), oral (Tang et al., 2017) and liver cancer (Roderburg et al., 2012). TNF-α has also been associated with the growth, proliferation, and immune evasion of tumour cells (Ben-Baruch, 2006).

**FIGURE 2:**
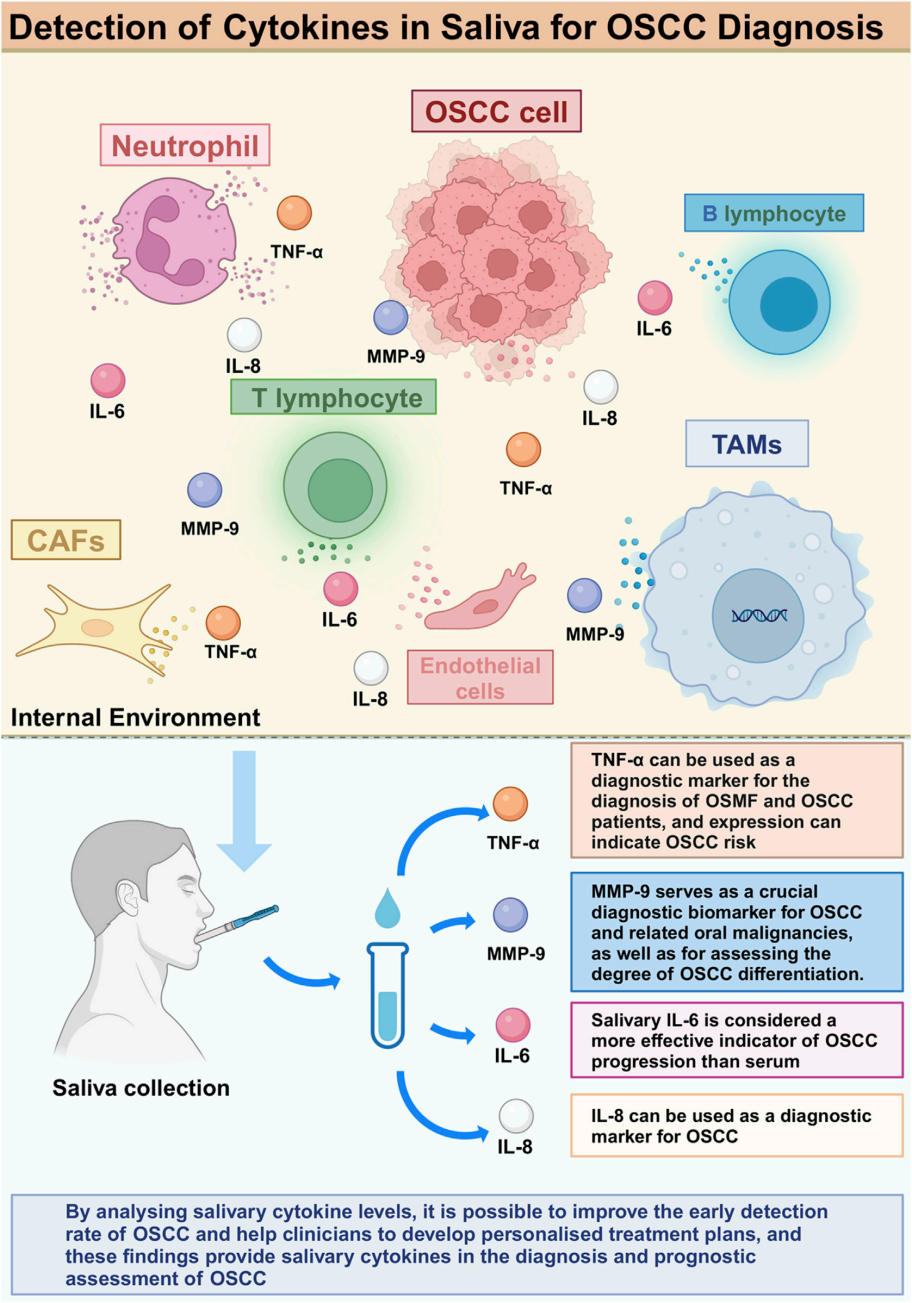
Detection of Cytokines in Saliva for OSCC diagnosis.

Increased levels of TNF-α were observed in saliva of patients with oral submucous fibrosis (OSMF) and OSCC. Abdul Aziz Shaikh et al. found that salivary TNF-α concentrations were significantly higher in patients diagnosed with OSMF stage 3, and the difference was statistically significant compared to patients in other stages (Abdul Aziz Shaikh et al., 2024) (Table 2). (Azimudin et al., 2024) (Table 2) suggested a potential role of TNF-α in OSCC, especially in diabetic patients, by suggesting that the mean level of TNF-α in saliva of diabetic (DM) patients and OSCC patients was significantly higher compared to that of the healthy population and OSCC patients without diabetes mellitus (Figure 2).

**TABLE 2:** Salivary cytokines associated with OSCC diagnosis and prognosis.

Biomarker	Discoveries	Clinical relevance	Reference
TNF-α	TNF-α concentrations are elevated in saliva of OSMF and OSCC patients and correlate with OSMF clinical stage	TNF-α expression may indicate risk of OSCC and can be used to indicate progression of OSCC	Abdul Aziz Shaikh et al. (2024)
TNF-α	Mean salivary TNF-α levels were higher in DM with OSCC compared to the healthy group and OSCC patients without DM	An in-depth look at the potential impact of TNF-α on OSCC (especially in diabetic patients)	Azimudin et al. (2024)
MMP-9	MMP-9 is associated with the staging of OSCC and other related malignant disease disorders	May be considered as a biomarker to differentiate the stage of pathogenesis and malignant transformation	Pazhani et al. (2023)
MMP-9	The expression of the cytokine MMP-9 is increased in both saliva and tumour tissue of patients	MMP-9 as a non-invasive saliva-based diagnostic and predictive biomarker	Cai et al. (2022)
IL-6	Salivary IL-6 is associated with OSCC precancerous lesions and its concentration increases with disease aggressiveness and severity	Salivary IL-6 may be a more effective indicator of OSCC progression than serum IL-6	Kalló et al. (2023), Naj et al. (2023), Oshin et al. (2024), Shree et al. (2024)

### 3.2 MMP-9

Matrix metalloproteinase-9 (MMP-9), also known as 92 kDa type IV collagenase, has an important role in tumorigenesis (Gobin et al., 2019). In various studies, the expression of MMP-9 has been shown to be an important component of oral cancer tissue (Patel et al., 2007), serum (Lotfi et al., 2015) and saliva (Shpitzer et al., 2009) diagnostic markers in the sample.

The secretion level of MMP-9 in saliva samples was correlated with OSCC differentiation. Pazhani et al. reported that salivary MMP-9 concentrations were significantly higher in OSCC patients, and were higher in poorly differentiated OSCC patients compared to highly differentiated and moderately differentiated OSCC patients. Increased amounts of this and other macromolecules have been identified in precancerous lesions linked to the advancement of OSCC, while salivary concentrations of MMP-9 are reported to be higher in patients with oral leukoplakia compared to those with OSCC. Elevated MMP-9 levels in the saliva of subjects with oral epithelial dysplasia are detectable even in mild and moderate instances, with an increase related to disease advancement. Salivary MMP-9 concentrations are notably higher in severe oral epithelial dysplasia, attributed to the increased likelihood of malignant transformation in these patients (Pazhani et al., 2023) (Table 2). Therefore, salivary MMP-9 can be used as a reliable indicator of malignant transformation.

Cai et al. found that the expression level of MMP-9 was not only increased in saliva, but also in tumour tissues of patients (Cai et al., 2022) (Table 2). Bioinformatics analysis showed that MMP-9 was associated with survival and tumour function in patients with head and neck cancer, suggesting that MMP-9 may be a saliva-based noninvasive diagnostic biomarker and a prognostic therapeutic target for OSCC.

### 3.3 IL-6

Interleukin 6 (IL-6) is important in several organs and systems, has a major impact on the immune response, is produced by cells such as epithelial cells and mast cells, and belongs to a major class of pro-inflammatory cytokines. When inflammation occurs, the concentration of IL-6 is usually elevated (Tanaka et al., 2014; Mauer et al., 2015; Hop et al., 2019; Kang et al., 2019). Increased salivary IL-6 levels have been documented in individuals with OSCC (Kalló et al., 2023; Naj et al., 2023; Oshin et al., 2024) (Table 2).

The level of IL-6 in saliva of OSCC patients has important diagnostic and prognostic significance. Shree et al. found a significant reduction in IL-6 expression after chemoradiotherapy (*p* < 0.0001), with significant changes in expression at different treatment intervals (Shree et al., 2024) (Table 2). This further confirms that IL-6 can be used as a biomarker for OSCC. It is present in precancerous lesions associated with OSCC and its pathogenesis (Naj et al., 2023; Oshin et al., 2024) (Table 2).

Moreover, the elevated levels of IL-6 are associated with the aggressiveness and severity of OSCC, leading to reduced survival and increased recurrence rates. It is noteworthy that salivary IL-6 is considered a more reliable marker of OSCC progression than serum IL-6 (Oshin et al., 2024) (Table 2). IL-6, mainly derived from macrophages, mediates the interaction among cells and is a very important OSCC-promoting cytokine in the tumour microenvironment, which makes salivary IL-6 an increasingly powerful biomarker for the diagnosis of OSCC.

### 3.4 Other cytokines

Interleukin-1β (IL-1β) plays a crucial role in immune response and inflammation, stimulates signaling pathways, promotes recruitment of inflammatory cells and is associated with a variety of diseases. Lee et al. (2018) concluded that the expression of this cytokine is elevated in both saliva and plasma of patients, particularly in the initial stages of OSCC, and its potential as a biomarker warrants greater consideration. Interleukin-8 (IL-8) is a significant chemokine with crucial immunological activities (Kim, 2020). Numerous publications have reported increased IL-8 levels in the saliva of patients diagnosed with OSCC (Csősz et al., 2017; Dikova et al., 2021). Other authors have also reviewed the value of IL-1β and IL-8 in saliva as biomarkers (Benito-Ramal et al., 2023; Kalló et al., 2023; Bastías et al., 2024; Khijmatgar et al., 2024). However, a large number of clinical trials are still required to further confirm their sensitivity and specificity as biomarkers in order to provide stronger evidence for early screening and personalized treatment of OSCC.

## 4 Discussion

Recent research indicates that assessing salivary miRNAs and cytokines is an effective approach for identifying and forecasting the outcomes of OSCC. These biomarkers facilitate the early detection of high-risk patients and furnish critical prognostic insights on the disease.

This review encapsulates the advancements of saliva-specific variables as biomarkers in the last 3 years, emphasizing the prospective significance of miRNAs and cytokines in OSCC. The significance of salivary miRNAs in diagnostic and prognostic applications has been a focal point of interest over the past 3 years, whereas there has been a relative scarcity of publications concerning salivary cytokines. Notably, the review mainly emphasized MMP-9, TNF-α, and IL-6, with IL-6 being specifically highlighted as a biomarker for OSCC. Studies related to IL-1, IL-8, and IL-10 were mostly concentrated in the last 3 years, while researches on diagnosis and prognosis were mainly performed before 2021, indicating that the role of cytokines as biomarkers has become more mature. But there is still a need to further optimize and standardize the relevant assays in order to improve the reliability of their clinical application. The amalgamation of several biomarkers is anticipated to improve the accuracy of early diagnosis and the efficacy of prognostic evaluation. In summary, salivary miRNAs and cytokines offer novel insights for the noninvasive diagnosis and personalized therapy of OSCC, warranting more investigation in the future.
